# Breast cancer, psychological distress and life events among young women

**DOI:** 10.1186/1471-2407-8-245

**Published:** 2008-08-22

**Authors:** Ronit Peled, Devora Carmil, Orly Siboni-Samocha, Ilana Shoham-Vardi

**Affiliations:** 1 Department of Health Systems Management, Faculty of Health Sciences, Ben-Gurion University of the Negev, Beer Sheva, Israel; 2 Center for the Study of Psychological Stress, Haifa University, Haifa, Israel; 3 Department of Epidemiology and Health Services Evaluation, Faculty of Health Sciences, Ben-Gurion University of the Negev, Beer Sheva, Israel

## Abstract

Since 1983, studies have suggested an interaction between the severe life events, psychological distress and the etiology of Cancer. However, these associations are still under dispute.

***The aim ***of the present study was to examine the relationship between life events, psychological distress and Breast Cancer (BC) among young women.

**Methods:**

A case control study. The study population included 622 women, under the age of 45 years. 255 were diagnosed for BC, and 367 were healthy women. A validated Brief Symptom Inventory (BSI) and Life Event Questionnaire were used.

**Results:**

The cases presented significantly higher scores of depression compared to the controls and significant lower scores of happiness and optimism. A significant difference was found when comparing the groups according to the cumulative number of life events (two or more events). A multivariate analysis suggest that exposure to more than one life event is positively associated with BC [Odds Ratio(OR) :1.62 95% Confidence Interval (CI): 1.09–2.40], and that a general feeling of happiness and optimism has a "protective effect" on the etiology of BC. (OR-0.75, 95% CI:0.64–0.86).

**Conclusion:**

Young women who were exposed to a number of life events, should be considered as a risk group for BC and treated accordingly.

## Background

Breast cancer (BC) is the most frequent malignant disease among women. According to American reports every third cancer is diagnosed as BC. In 2002 the incidence rate and death rate for invasive BC, were 124.9 and 25.5 per 100,000 women respectively [1]. In Europe breast cancer has become the commonest cancer diagnosed overall, with 429,900 new cases in 2006 (13.5% of all cancer cases), before lung cancer [2].

In Israel BC rates are among the highest in the world. During the last 25 years a steady increase in the incidence rates has been documented. in 2002, the age adjusted incident rates for women aged 25 – 49 and 65+, were 159.63 and 350.13 per 100,000 women, respectively [3].

Several risk factors have been documented in the scientific literature, among them are: family history [4], radiation exposure [5], androgenic estrogens [6-8], nutrition and diet habits [9,10], smoking, alcohol consumption, lack of physical activity and lack or short term of breast feeding [11] and social status [12]. However, it was estimated that these factors explain only 40% of the BC cases [13].

The relationship between "body and mind" is an old issue. In 1893 HL Snow reported that 150 out of 200 BC cases, experienced a traumatic life event, associated with the loss of a close relative, prior to diagnosis [14]. Since then, numerous scientific publications documented the relationship between stressful life events, depression and/or anxiety and the development of the disease [15-20]. In 2005 Glaser and Kiecolt in their review of experimental in- vivo and in -vitro studies, explained how psychological factors are associated with immune dysfunctions and the development of malignant cells. These studies took into account the type and severity of the event, the effect of accumulated events and the burden of the psychological distress developed by the event. These series of studies provide some evidence of the pathways through which psychological stress could contribute to the increase of cancer risk [21].

However, despite the fact that some empirical evidence regarding the interaction between the endocrine system, psychological distress and the etiology of the disease, has been suggested, the subject is still under dispute. It is also probable that personality characteristics and personal resources, as well as coping skills and social support are playing an important role in these associations [22].

*The main objective *of the present study was to examine the relationship between life events, psychological distress and BC among young women.

*We hypothesized *that psychological distress and severe life events are risk factors for BC among young women. We also *hypothesized *that there is a cumulative effect of life events on the initiation of the disease.

## Methods

A case – control study was designed.

### Study Population

**Cases **were women aged 25 – 45 who had been diagnosed for BC between 1998–2002. The diagnosis was based on histological tests, carried out in six major oncology units in Israel. Letters were sent to the women by their physicians asking them to take part in the study. Those who agreed and have signed an inform consent, were interviewed. The response rate was 25%. The interviews were carried out in 2002 and the mean time between the time of diagnosis and the interview was one year (median- seven months). No data was available to compare the responders to the non responders.

**Controls **were women aged 25–45 who visited the out patient clinics of Shiba Medical center, the largest hospital in central Israel and Barzilai Medical Center, a relatively small hospital located in the southern region of the country. The inclusion criteria was being free of BC and or other malignant disease. They were interviewed in 2004 after signing an inform consent. We assume that the two groups are quite similar since they were recruited from the same type of medical centers with the same referral and health insurance patterns.

### Tools

1) A demographic questionnaire 2) A questionnaire measuring psychological distress: A short version of 15 items of the Brief Symptom Inventory (BSI) [23,24] which has an Hebrew validated version [25]. The questionnaire included six items evaluating depression, six items evaluating anxiety, and three items evaluating happiness and optimism. 3) Life event questionnaire included 10 events [26]. The list of the severe life events included four items: A) a loss of a parent before twenty years of age B) divorce of parents before twenty years of age C) a loss of a close relative D) a loss of a spouse. The list of mild to moderate life events included six items: A) a separation from a spouse, B) a loss of a job, C) an economic crisis D) a severe illness of a close relative E) a severe illness F) other moderate event.

Women in both groups were given two separate lists with no directions which of them is considered as "severe" or "moderate" and were asked about age at exposure. Study cases were asked about their feelings and life events exposure prior to their diagnosis.

*Analysis of data *was carried out by SPSS software. For univariante analysis we used Chi Square, t-Test and Correlation Tests according to the nature of the variables.

Statistical significance was considered as P ≤ 0.05. For multivariate analysis logistic regression models were estimated for the dependent variable: case/control in two steps. In the first step each event was added to the model separately as well as the independent variables which were considered as statistical significant in the univariant analysis: age and family status. In the second step and in order to measure the effect of accumulated life events, two dummy variables were built out of the ten events ; for one event and for more than two events. In this case "no events" served as reference. In both steps depression, anxiety and happiness were represented by their mean scores. The final model included variables which were significant in the univariante analysis and in the preliminary models, Hosmer and Lemeshow goodness of fit test was used to compare the models and choose the best one [27]. In all models Odds Ratios and 95% Confidence Intervals were calculated.

### Ethics

The study was approved and supervised by the Local Ethical Committee according to the principles embodied in the Declaration of Helsinki.

## Results

Two hundred fifty five cases, and three hundred sixty seven controls were recruited. The mean age was 40 (± 4.8) and 34.7 (± 6.3) years for cases and controls respectively (P < 0.001). Differences in marital status between the study groups were statistically significant (Table 1). No significant differences were found in mean age when life event was experienced between the study groups only in "loss of close relative". In this case the control group was younger than the cases (Table 2).

**Table 1:** Demographic Characteristics

	***Cases***	***Controls***	***P***
***Mean Age (SD)***	40.03 (4.8)	34.77 (6.3)	<0.001
***Education: % > 12 years***	54.2	31	0.505
***Marital Status: % married or living with a spouse***	85.1	75	0.002

**Table 2:** Distribution of life events and mean age at experience, by study groups (%)

	**Cases**		***Controls***		***Events***	***Age***
**Severe life events**	**%**	**Age**	**%**	**Age**	***P***	***P***

**Loss of a close relative**	51.9	28.0	44.5	25.5	0.070	0.042
**Loss of a spouse**	1.7	28.0	6.4	25.2	0.189	0.841
**Parents divorce before age 20**	9.7	No data	6.4	No data	0.122	--
**Loss of a parent before age 20**	10.1	No data	7.5	No data	0.192	--
***Moderate to mild events***						
**Spouse separation**	19.8	29.0	18.6	29.0	0.714	0.655
**Job loss**	22.7	32.6	18.0	32.6	0.181	0.548
**Economic crisis**	14.3	33.5	15.9	33.5	0.607	0.418
**Severe illness**	5.1	29.9	7.8	29.9	0.173	0.815
**Severe illness of a close relative**	32	32.0	30.1	32.0	0.459	0.322
**Other severe event**	31.2	30.8	20.7	30.8	0.003	0.555
**Having one event**	26.8	No data	33.6	No data	0.094	--
**Having two and more events**	52.0	No data	43.9	No data	0.065	--

The cases presented significantly higher scores of depression compared to the controls and significant lower scores of happiness and optimism. Both groups, but mostly the cases, were found to have higher scores of depression and anxiety compared to the Israeli standards (Table 3).

**Table 3:** Mean scores for psychological distress parameters

	***Cases***	***Controls***	***P***	***Israeli Standard †***
Anxiety*	1.32	1.27	0.425	0.85
***(SD)***	(0.8)	(0.7)		
Depression*	1.02	0.89	0.04	0.70
***(SD)***	(0.8)	(0.7)		
Happiness & Optimism**	4.9	5.4	0.00	--
***(SD)***	(1.5)	(1.2)		

† Gilbar and Ben Zur 2002(^22^).

* Scale: 0 (Low) – 4 (High).

** Scale: 1 (Low) – 7 (High)

No significant differences were found between the study groups with regard to the distribution of specific life events. However. a significant difference was found when comparing the groups according to the cumulative number of events (two or more events) (Table 2).

The results of the final regression models suggest several factors that are associated with the disease: age (this is an artifact of the study design and should be controlled for. Yet, it can also be regarded as a "risk factor"), marital status (being married or living with a spouse) and exposure to more than one life event (OR :1.62 95% CI: 1.09–2.40) (Table 4). It was also found that a general feeling of happiness and optimism has a "protective effect" on the etiology of BC. (OR-0.75 95% CI: 0.64–0.86).

**Table 4:** The results of the final regression model

***Variable***	***B***	***SE***	***P***	***OR***	***95% CI***
***Marital Status (married vs. single)***	0.617	0.26	0.019	1.85	1.10–3.10
***Happiness & Optimism***	-0.291	0.075	<0.0001	0.75	0.64–0.86
***Age***	0.152	0.019	<0.0001	1.16	1.12–1.20
***Two and more events (vs. all others)***	0.483	0.20	0.017	1.62	1.09–2.40

OR = Odds Ratios, 95CI = 95% Confidence Interval

Hosmer and Lemeshow test = 0.306 chi-square = 9.441. – 2 log likelihood = 612.1

## Discussion

The main results of our study suggest a negative association between happiness, optimism and BC and a positive association between depression, life events and the disease. As expected, general feeling of happiness was negatively correlated with depression. We could not demonstrate a positive association with each separate life event. Yet, exposure to a cumulative number of events (more than one) was positively associated with the disease. In other words, we can carefully say, that experiencing more than one meaningful life event (severe and/or mild to moderate) is a risk factor for breast cancer among young women. On the other hand, general feelings of happiness and optimism can play a protective role against the disease. At this point we should mention again, that the cases were interviewed after they were diagnosed for BC, and although they were asked to report about their feelings prior to diagnosis, the illness might have effected the way they evaluate their life in general.

Little is known about the role that stress plays as a cofactor, in either the initiation of cancer or the progression of a tumor once developed. Yet, several epidemiological studies conducted in the early nineties, demonstrated relationships between psycho-social factors, such as life events, social support and social networks, self efficacy, defensive behavior and breast cancer [28,29]. Other studies presented relationships between BC and meaningful life events such as a loss of a spouse, a close relative or a close friend [16-18]. However, Studies that attempt to link psychological stressors with the initiation or progression of cancer are difficult to perform. The main problem lies in the retrospective nature of such studies, as well as the many cofactors involved.

A series of experimental studies provide some evidence of the process through which psychological stress could contribute to the increase of the risk of cancer, by modifying cell responses to environmental factors. At the same time, the mechanism in which the central nerve, endocrine and immune systems interact and how behavior, and or external events modulate these three complex systems in not fully understood [21,30,31].

The scientific literature about stress is also pre-occupied with the question, whether coping with a severe life event protects us against the effect of mild events. As mentioned earlier, when we examined the effect of each event on the development of BC, we could not demonstrate a significant result however, exposure to a number of events (severe or mild to moderate) was significantly associated with the disease. This suggests that stressful events do not protect us from the effect of additional events, and even "moderate or mild" events, seem to have a cumulative effect.

Our results did not show a significant association with anxiety, although the study group (sick women) exhibited higher scores of anxiety compared to the controls, and to the Israeli standards [23]. We suggest that future studies should investigate the different relationship between anxiety and depression with disease in general and malignant disease in particular.

As previously stated, general feelings of happiness and optimism seems to play a protective role against the disease. Although lately research studies have started to examine the relationship between happiness and health, there is no evidence in the literature to such a relationship with BC. Certain support can be found in a study by Kim and colleagues [32] reporting a negative relationship between positive life events and the development of BC.

Some limitations of the present study should be pointed out. The study population can not be considered a representative sample of the relevant population. The response rate among cases was low (25%) and the controls were not randomly selected. Regarding the questions about the general feelings of happiness and satisfaction in life, as described earlier, we asked the study group about their feelings prior to diagnosis. The average lag of time between the diagnosis and the interview was one year, and although we do not consider a recollection bias, the fact that the women knew about their illness when interviewed, could have affected their answers. Barraclough J in his letter from 1996 to the BMJ discusses this unavoidable problem in retrospective studies in which the patient is over reporting of stress in an effort to explain the illness or due to the knowledge of having cancer [33]. Yet, in our study, while the stress report might be biased, the adverse life events: a loss of a parent and/or a close relative, and or a divorce or loss of a spouse can be objectively verified and dated as suggested by Barraclough.

### What this paper adds

▪ Our study offers additional support to the knowledge regarding the interaction between psychological distress, traumatic life events and the development of cancer. Although there are some evidence of the association between negative and or positive life events and cancer, this important scientific question needs further investigation.

▪ Our study sheds light on the cumulative effect of life events on the etiology of BC.

▪ Our study offers some precocious suggestions about the protective role happiness and sense of optimism are playing in the development of BC.

▪ From a policy making point of view, we suggest that young women who suffered a loss in their early childhood, especially those exposed to a number of life events, should be considered as a risk group and be treated accordingly.

▪ The relationship between happiness and health should be examined in future studies and possible relevant preventive initiatives should be developed.

## Competing interests

The authors declare that they have no competing interests.

## Authors' contributions

RP was the principal investigator of this study responsible for the design and statistical analysis. DC designed and supervised the tools for data collection. OS–S carried out the data collection and literature review and participated in the statistical analysis. IS–V supervised the design and statistical analysis.

## Pre-publication history

The pre-publication history for this paper can be accessed here:



## References

[B1] US Cancer Statistics http://apps.nccd.cdc.gov/uscs/.

[B2] WHO International Agency for Research on Cancer. http://www.iarc.fr/en/Media-Centre/IARC-Press-Releases/Recent-Releases/New-European-cancer-figures-World-Cancer-Agency-says-major-efforts-needed-toward-prevention-in-Europe.

[B3] Israeli MOH http://www.health.gov.il/download/pages/breast_young_march_2003.pdf.

[B4] King MC, Marks JH, Mandell JB (2003). Breast and ovarian cancer risks due to inherited mutations in BRCA1 and BRCA2. Science.

[B5] Yankaskas BC (2006). Epidemiology of breast cancer in young women. Breast Dis.

[B6] Hulka BS, Stark AT (1995). Breast cancer: cause and prevention. Lancet.

[B7] Hulka BS, Moorman PG (2001). Breast cancer: hormones and other risk factors. Maturitas.

[B8] Kuller L, Cauley J, Lucas L (1994). Quality Determinants of Mammography.

[B9] Schernhammer ES, Laden F, Speizer FE, Willett WC, Hunter DJ, Kawachi I, Colditz (2001). Rotating night shifts and risk of breast cancer in women participating in the nurses' health study. J Natl Cancer Inst.

[B10] Cui Y, Miller AB, Rohan TE (2006). Cigarette smoking and breast cancer risk: update of a prospective cohort study. Breast Cancer Res Treat.

[B11] Clarke CA, Purdie DM, Glaser SL (2006). Population attributable risk of breast cancer in white women associated with immediately modifiable risk factors. BMC Cancer.

[B12] Kelsey JL, Horn-Ross PL (1993). Breast cancer: magnitude of the problem and descriptive epidemiology. Epidemiol Rev.

[B13] Bleiker EM, Ploeg HM van der (1999). Psychosocial factors in the etiology of breast cancer: review of a popular link. Patient Educ Couns.

[B14] Snow HL (1893). Cancer and the cancer process.

[B15] Jacobs JR, Bovasso GB (2000). Early and chronic stress and their relation to breast cancer. Psychol Med.

[B16] Duijts SF, Zeegers MP, Borne BV (2003). The association between stressful life events and breast cancer risk: a meta-analysis. Int J Cancer.

[B17] Kruk J, Aboul-Enein HY (2004). Psychological stress and the risk of breast cancer: a case-control study. Cancer Detect Prev.

[B18] Ollonen P, Lehtonen J, Eskelinen M (2005). Stressful and adverse life experiences in patients with breast symptoms; a prospective case-control study in Kuopio, Finland. Anticancer Res.

[B19] Forsén A (1991). Psychosocial stress as a risk for breast cancer. Psychother Psychosom.

[B20] Lillberg K, Verkasalo PK, Kaprio J, Teppo L, Helenius H, Koskenvuo M (2003). Stressful life events and risk of breast cancer in 10,808 women: a cohort study. Am J Epidemiol.

[B21] Glaser R, Kiecolt-Glaser JK (2005). Stress-induced immune dysfunction: implications for health. Nat Rev Immunol.

[B22] Baltrusch HJ, Stangel W, Titze I (1991). Stress, cancer and immunity: new developments in biopsychosocial and psychoneuroimmunologic research. Acta Neurol (Napoli).

[B23] Derogatis LR, Melisaratos N (1979). The brief symptom inventory: administration, scoring and procedures. Manual 1 Clinical psychometric research.

[B24] Derogatis LR (1982). The BSI administration, scoring and procedures. Manual 2 Clinical psychometric research, Baltimore.

[B25] Gilbar O, Ben-Zur H (2002). Bereavement of spouse caregivers of cancer patients. Am J Orthopsychiatry.

[B26] Schwartz R, Geyer S (1984). Social and psychological differences between cancer and non cancer patients :cause or consequence of the disease?. Psychother Psycosom.

[B27] Hosmer DW, Lemeshow S (1989). Applied Logistic Regression.

[B28] Geyer S (1991). Life events prior to manifestation of breast cancer: a limited prospective study covering eight years before diagnosis. J Psychosom Res.

[B29] Chen CC, David AS, Nunnerley H, Michell M, Dawson JL, Berry H, Dobbs J, Fahy T (1995). Adverse life events and breast cancer: case-control study. BMJ.

[B30] Abercrombie HC, Giese-Davis J, Sephton S, Epel ES, Turner-Cobb JM, Spiegel D (2004). Flattened cortisol rhythms in metastatic breast cancer patients.

[B31] Turner-Cobb JM, Sephton SE, Koopman C, Blake-Mortimer J, Spiegel D (2000). Social support and salivary cortisol in women with metastatic breast cancer. Psychosom Med.

[B32] Kim Y, Duhamel KN, Valdimarsdottir HB, Bovbjerg DH (2005). Psychological distress among healthy women with family histories of breast cancer: effects of recent life events. Psychooncology.

[B33] Barraclough J (1996). Adverse life events and breast cancer. BMJ.

